# Temporal Study of the Microbial Diversity of the North Arm of Great Salt Lake, Utah, U.S.

**DOI:** 10.3390/microorganisms3030310

**Published:** 2015-07-02

**Authors:** Swati Almeida-Dalmet, Masoumeh Sikaroodi, Patrick M. Gillevet, Carol D. Litchfield, Bonnie K. Baxter

**Affiliations:** 1 Microbiome Analysis Center, Department of Environmental Science and Policy, George Mason University, 10900 University Blvd., Manassas, VA 20110, USA; E-Mails: sdalmet@gmu.edu (S.A.-D.); msikaroo@gmu.edu (M.S.); pgilleve@gmu.edu (P.M.G.); gslinstitute@westminstercollege.edu (C.D.L.); 2 Great Salt Lake Institute, Westminster College, 1840 South 1300 East, Salt Lake City, UT 84105, USA

**Keywords:** molecular phylogeny and molecular biology, haloarchaea, halophile: ecology, biotechnology, phylogeny, genetics, taxonomy, enzymes

## Abstract

We employed a temporal sampling approach to understand how the microbial diversity may shift in the north arm of Great Salt Lake, Utah, U.S. To determine how variations in seasonal environmental factors affect microbial communities, length heterogeneity PCR fingerprinting was performed using consensus primers for the domain *Bacteria*, and the haloarchaea. The archaeal fingerprints showed similarities during 2003 and 2004, but this diversity changed during the remaining two years of the study, 2005 and 2006. We also performed molecular phylogenetic analysis of the 16S rRNA genes of the whole microbial community to characterize the taxa in the samples. Our results indicated that in the domain, *Bacteria*, the *Salinibacter* group dominated the populations in all samplings. However, in the case of *Archaea*, as noted by LIBSHUFF for phylogenetic relatedness analysis, many of the temporal communities were distinct from each other, and changes in community composition did not track with environmental parameters. Around 20–23 different phylotypes, as revealed by rarefaction, predominated at different periods of the year. Some phylotypes, such as *Haloquadradum*, were present year-round although they changed in their abundance in different samplings, which may indicate that these species are affected by biotic factors, such as nutrients or viruses, that are independent of seasonal temperature dynamics.

## 1. Introduction

Great Salt Lake is bisected by a railroad causeway, which creates virtually two lakes, each with distinct environmental conditions [1]. The south arm of this terminal lake ranges from 11% to 15% salinity [2] as it receives fresh water input from the runoff of snowmelt in the Wasatch mountain range. However, the isolated north arm has little fresh water input, save precipitation. Consequently, the north arm salinity is often at saturation (up to 30%).

Due to the nature of a terminal lake with no outflow, Great Salt Lake is subjected to extreme variations in environmental conditions. The elevated desert biome results in seasonal variations of water temperature ranges from 0.5 °C in January to 26.7 °C in July [3] and up to 45 °C in the shallow margins [4]. These in turn contribute to differential maximum solute concentrations and rates of evaporation. Variable precipitation in contributing watersheds caused the lake to rise four meters from 1983 to 1987, and six years of drought left the lake at a historic low in 2004 (until spring 2005), which has since returned to average depth. Brine stratification plays a role in the local conditions and, depending on depth and weather, mixing and turnover will alter stratification unpredictably [2]. Since Great Salt Lake is a dynamic ecosystem, we sought to address the question of changes that occur over time in the microbial communities of the isolated north arm.

Studies on temporal dynamics serve to explore the connection between microbial population structure and environmental parameters. Understanding how communities shift in a changing environment allows one to predict the presence of particular microbial processes [5] or the species composition [6]. Microbial community profiles over time may display a cyclical or trajectory pattern, or they may be more stable with shared taxa (reviewed in [7]). The types and numbers of microorganisms in a particular habitat are a function of a variety of factors including available nutrients and an interplay of environmental conditions that act as selective forces [8]. An often-overlooked factor that can moderate microbial community profiles is the presence of viruses, which can shift the genera or species present based on specific predator/prey relationships. In the north arm of Great Salt Lake, a broad diversity of virus particles is present and outnumbers bacteria and archaea cells by 100-fold [9]. Changes in communities are likely the consequences of the interactions among both biological and environmental factors [10], but which are the most significant for Great Salt Lake?

Molecular tools allow us to identify new phylotypes and follow shifts in microbial communities [11,12,13]. However, there are only a few such studies on the microbial diversity of Great Salt Lake [14,15,16,17]. These are “snapshot” studies that look at genetic or metabolic diversity with a spatial context. None of these approaches were designed to address temporal changes in the community profiles. This taken together with the paucity of information on the seasonal dynamics of the north arm of Great Salt Lake inspired us to begin an intensive examination of the microbial community at Rozel Point in the Fall of 2003, incorporating both culture-based and molecular analyses. To our knowledge, this is the first study to provide insight into the temporal distribution of microbial diversity in this extreme environment.

## 2. Experimental Section

### 2.1. Sample Collection

All samples were taken from the shallow shoreline at Rozel Point in the north arm of Great Salt Lake. The sampling site is located 41°26.285′ N and 112°40.062′ W and at the altitude of 1265.4 m. The exact locations were recorded by GPS and varied only slightly depending on the season due to changing water levels and shoreline locations. The specific dates and environmental conditions at the time of sampling are shown in Table 1. Since the regional temperature of the area of sampling is critical to salinity, dissolved oxygen (DO), and other parameters, we employed satellite imagery data for temperature measurements of the exact location and time [3]. The salinity was recorded with refractometer from SPER Scientific (Scottsdale, AZ, USA). The record on the elevation of the lake was obtained from the website [18]. June and October were chosen as annual high and low points in this cycle since June follows the spring runoff of the Wasatch Mountains and October follows the typical Utah dry, desert summer. We had the opportunity to sample one year in February, which served as a marker for both low temperature and mid-level water input. The roads to the sampling site are often impassable in winter, making winter month sampling very challenging. We decided to include this “opportunistic sampling” to draw a larger picture with the caveat that we were only able to do it one winter.

**Table 1:** Characterization of Great Salt Lake north arm samples.

Sample Name	Sampling Date	pH	DO mg/L	^§^ Temperature in °C	^£^ Lake Elevation (Meters)	Salinity %	Cell Enumeration (CFUs)/Ml *
NA1	JUN2003	7.62	1.36	23	1279.18	29	4 × 10^6^
NA2	OCT2003	7.62	1.17	18	1278.66	27	6.5 × 10^6^
NA4	JUN2004	7.62	1.36	22	1278.69	30	1.1 × 10^3^
NA6	FEB2005	7.62	ND	3	1278.57	24	4 × 10^5^
NA7	JUN2005	7.62	1.36	20	1279.00	29	3.5 × 10^6^
NA9	JUN2006	7.62	1.36	22	1279.24	30	3.5 × 10^4^
NA10	OCT2006	7.62	1.17	18	1278.84	28	2 × 10^3^

ND—Not determined ^§^ (Crosman and Horel 2009 [3]) ^£^ Great Salt Lake-Lake Elevations and Elevation Changes [18] elevation above National Geodetic Vertical Datum 1929 (NGVD, 1929). * mean of the triplicate platings.

The brine depth in this hypersaline part of the lake was approximately one meter in accessible locations, therefore, only one subsurface sample was taken at each sampling time. The brine samples for all experiments were collected aseptically and used directly for cultivation and enumeration. A portion of each sample (4–6 liters) was centrifuged to obtain a cell pellet which was frozen at −20 °C or −80 °C until used for DNA extraction. Additional samples were used directly for cultivation and direct cell counts using a hemocytometer (data not shown).

### 2.2. Cultivation and Enumeration

We inoculated media with the brine samples immediately after collection at each sampling time. In order to cover a wide range of potential nutrient requirements, two different media were used at four NaCl concentrations: 8%, 10%, 15%, and 25% (w/v) solar salt (Cargill, Minneapolis, MN, USA). One of the media was modified casamino acid medium (MCAT) [19,20] For the other medium, R2A medium (Difco, Franklin Lakes, NJ, USA) was modified by the addition of 20 g/L magnesium sulfate and was also used at the above solar salt concentrations[21]. Since modified R2A allowed much higher colony counts, we used it for reporting in this study. Decimal dilutions were prepared in isotonic solar salt water, surface spread-plated in triplicates onto the above media, and incubated at ambient temperatures. The colony-forming units (CFU) were counted after six weeks, and the data are reported here as CFU/mL of the original brine sample.

### 2.3. Whole Community Fingerprinting

The DNA was extracted from the cell pellets with the FastDNA Spin Kit for Soil (MP Biomedicals, Solon, OH, USA) following the manufacturer’s instructions. Aliquots of the extracted DNA were fingerprinted using the length heterogeneity polymerase chain reaction (LH-PCR) [22]. In this method, a fluorophore was added to the 5′ end of the forward primer, and the resulting LH-PCR products were separated on the SCE9610 capillary fluorescent sequencer (Spectrumedix LLC, State College, PA, USA) and analyzed with the GenoSpectrum™ software package (Version 2.01, State College, PA, USA). Details of our techniques have been published [20,23]. Briefly, for the domain *Bacteria*, the universal primers 27F (6-FAM-5′-AGAGTTTGATCMTGGCTCAG-3′) and 355R (5′-GCTGCCTCCCGTAGGAGT-3′) were used [24]. For the domain *Archaea*, the consensus primers 1HKF (6-FAM-5'-ATTCCGGTTGATCCTGCCGG-3′) and H589R (5′AGCTACGGACGCTTTAGGC-3′) were used [23]. With either set of 0.25 mM primers (PE Biosystems, Foster City, CA, USA), the PCR mixture contained 1X PCR buffer, 0.25 mM MgSO_4_, 2.5 mM dNTPs, 0.25U Tfl DNA polymerase, 5–40 ng DNA template, and DEPC-treated water to final volume of 20 μL per reaction. The DNA of pure culture of *Archaea* (*Haloferax volcanii*) and *Bacteria* (*E. coli*) were used as positive control and diethyl pyrocarbonate (DEPC) treated water was used as negative control. The PCR was performed in a PTC-100 programmable Thermal Cycler (MJ Research, Watertown, MA, USA).

For the *Bacteria*, the thermocycler conditions were: Initial denaturation at 95 °C for 5 min then 29 cycles of 94 °C (1 min), 48 °C (1 min), 72 °C (2 min), with a final extension at 72 °C (30 min) and hold at 4 °C. For the *Archaea* the thermocycler conditions were: Initial denaturation at 95 °C for 5 min, then 25 cycles of 94 °C (1 min), annealing at 55 °C (1 min), extension at 72 °C (3 min) with final extension at 72 °C (30 min) and hold at 4 °C. When nonlabeled primers were used, they were purchased from Invitrogen (Gaithersburg, MD, USA).

### 2.4. Cloning and Sequencing

In order to analyze the 16S rRNA genes from each sampling, we produced clone libraries for analysis. For cloning and sequencing the same PCR conditions described above were used, except that the primers (Invitrogen, Gaithersburg, MD, USA) were not labeled with 6-FAM, and the final extension step was 10 min. The fresh PCR products were cloned using chemically competent cells and the TOPO TA™ cloning kit (Invitrogen, Gaithersburg, MD, USA). Kanamycin (10 mg/mL) was added to the ImMedia plates (Invitrogen, Gaithersburg, MD) to select transformed cells. For each clone library approximately 80–90 white colonies were randomly picked into a 96 well microtiter plate containing 50 μL of Tris-EDTA buffer at pH 8 and lysed at 95 °C for 10 min. The microtiter plates were centrifuged for 5 min at 2000 rpm to precipitate the cell debris. The DNA in the supernatant was amplified with universal M13 primers (Invitrogen, Gaithersburg, MD, USA) and Taq Gold polymerase (Promega, Madison, WI, USA) with the following conditions: Initial denaturation at 95 °C (11 min), and 40 cycles of denaturation at 95 °C (30 s), annealing at 45 °C (30 s), then 72 °C (2 min) with 5 s extension per cycle. After 40 cycles of steps 2–4, the final extension was at 72 °C (10 min) and it was hold at 4 °C. The PCR product was visualized with ethidium bromide on a 1% agarose gel and then was purified using Agencourt AMPure (Beckman Coulter Inc., Brea, CA, USA) solution. The Big Dye Terminator Kit (Life technologies, Grand Island, NY) was used with GeneAmp PCR system 9700 (Applied Biosystems) for the standard sequencing reaction, and the product was purified using the Sephadex G-50 gel filtration system (Sigma-Aldrich, St. Louis, MO, USA). The product was then dried in a Speedvac (Savant AES 2010), and kept at −20 °C until it was reconstituted in Hi-Di Formamide (Applied Biosystems) to run on the SpectruMedix SCE 9610 (SpectruMedix LLC) capillary sequencer.

### 2.5. Phylogenetic Analysis

The archaeal and bacterial 16S rRNA gene sequences were aligned into contigs using Sequencher software v4.7 (Gene Codes Corporation, Ann Arbor, MI, USA) to trim off the primer sequences and manually correct ambiguities when needed. Clone sequences were analyzed by Basic Local Alignment Search Tool (BLAST) in GenBank [25] to obtain the sequences of the closest relative. The web-based Bellerophon [26] was used for the identification of chimeric sequences, and those sequences were discarded. Then the gene sequences were imported again into Sequencher software v4.7 along with reference sequences from GenBank. The sequences were realigned using Clustal X [27]. Since shorter sequences do not provide much information, only sequences longer than 200 bases were used for the construction of the phylogenetic tree. The aligned sequences were then exported to PAUP [28] to construct the neighbor-joining phylogenetic tree. *Desulfurococcus fermentans* and *Desulfurococcus sacchrovorans*, hyperthermophilic *Archaea* were used as the out-groups and the robustness of the tree was estimated by bootstrap resampling of the neighbor joining tree. The bootstrap values were calculated for 1000 replicates. The values greater than 70 are shown at the branch points.

### 2.6. Statistical Analysis

To assay the significance of the different Great Salt Lake communities sampled over time, we employed the LIBSHUFF software v0.96 [29], which is designed to compare two libraries of 16S rRNA gene sequences [30]. This analysis was used for comparing the clone libraries of each sampling. Homologous coverage denotes the predicted coverage of a sampled library and the heterologous coverage is the observance of similar sequence in a separate library. If the two samples are significantly different, the homologous coverage curve and the heterologous coverage curve will differ. When more than two libraries were compared, Bonferroni correction was applied. The abundances for archaea were plotted against the sampling period. The abundances were obtained by using the RDP10 Bayesian classifier in our custom Galaxy portal [31].

Rarefaction curves were calculated to estimate the minimum number of clones needed to ensure maximum coverage of the sample diversity using Analytical Rarefaction software v1.3 [32]. Abundances for the rarefaction were obtained by using a custom Perl script [23]. To define the Operational Taxonomic Unit (OTU), 95% cutoff was used and to interpret rarefaction curves upper 95% confidence intervals were used. Standardized environmental data (Table 1) were used for correlation analysis of environmental variables and canonical correspondence analysis. These analyses were done using Microsoft Excel 2013 and Multivariate Satistical Package v3.1 (Kovach computing services, Pentraeth, Wales, UK). The significance level was set at *p* < 0.05.

### 2.7. Accession Number

The archaeal 16S rRNA gene sequences were deposited in the GenBank database under the accession numbers JX438191 through JX438276 and JX668230 through JX668687. The bacterial 16S rRNA gene sequences were deposited in the GenBank database under the accession numbers KF569484 to KF569486.

## 3. Results

### 3.1. Characterization of the Microbial Communities of the North Arm of Great Salt Lake

A terminal lake experiences cycles of drought and flooding, and Great Salt Lake is not an exception. This cycle influences the salinity and nutrient concentrations of the waters as high precipitation can dilute the brine, and the lake experiences temperature fluctuations as a high desert, shallow body of water. For these reasons, we hypothesized that we would observe shifts over time in numbers of cells and the profiles of species in the local microbial community of the north arm.

To assay for cell number, we plated brine samples and observed colony-forming units (CFU). In this study, the numbers of CFUs counted ranged from 10^3^ to 10^6^, with the lowest number observed in June 2004 and October 2006 (Table 1). The number of cells in the water column clearly changed between samplings, but they had no relationship with temperature or season.

**Figure 1:**
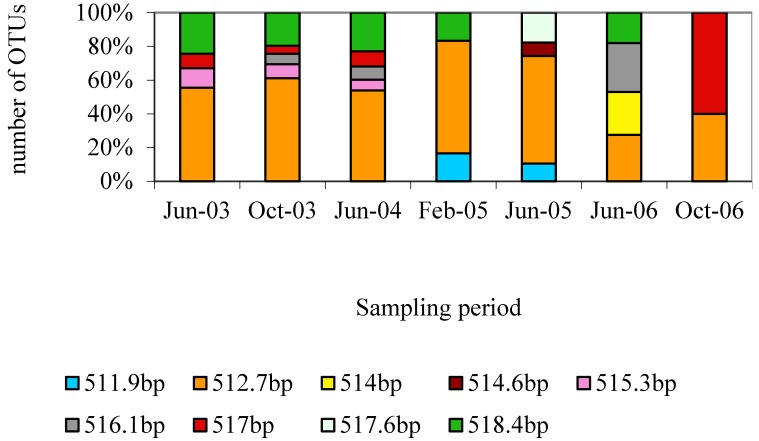
Amplicon Length Heterogeneity fingerprints of the archaeal 16S rRNA gene fragments. The Y-axis shows the percentage representation (relative abundance) of each OTU (operational taxonomic unit) had in the population. Each OTU length is given in base pairs and is indicated by a separate color (see legends).

To assess the change in the structure of Great Salt Lake microbial community at different times, Length-Heterogenity (LH)-PCR fingerprinting was used. LH-PCR fingerprinting is based on use of natural variation in the length and base composition of variable regions of 16S rRNA gene. Each peak or length represents an Operational Taxonomic Unit (OTU), which may be a strain, species or genus. It should also be noted that a particular peak might represent more than one taxon. Amplicons were analyzed for length in base pairs, and different lengths represent diverse representation (OTUs) from the microbial population. These OTUs are relative abundances of amplified sequences obtained from different organisms. For the domain *Bacteria* only a single OTU (~350 bp) was observed (data not shown). In contrast, results for the domain *Archaea* showed a fairly diverse community (Figure 1). This coincides with our expectation that we would observe more archaeal community members than bacterial, given the extreme salinity of saturated brine.

Figure 1 shows the distribution of the OTUs for archaea throughout the course of this study. The OTU pattern was similar from June 2003 until June 2004. However from February 2005 the pattern changed randomly and new OTUs appeared in every sampling. We considered that the reason behind this shift could be the low lake elevation (1278.57 m) in February 2005 (Table 1). Both the decreased volume of water and the temperature affect salinity, which could be a driving factor. The OTU_512.7 was common and one of the major peaks in all the samples. Similarly OTU_518.4 was the other major peak found in all samples with the exception of June 2005, and October 2006.

### 3.2. Identification and Phylogenetic Analysis of the Temporal Diversity of Microbial Communities

GenBank analysis revealed that almost all of the sequences of the bacterial library belonged to the family *Rhodobacteraceae* of which 98% belonged to the genus *Salinibacter*, very rare *Halomonas* and a few belonged to uncultured *Bacteria*. Therefore phylogenetic analysis was not done on the bacterial clones.

For each archaeal clone library, 80–90 sequences were analyzed. The chimeric sequences were checked with Bellerophon analysis and around 70 chimeric sequences out of a total of 600 sequences were discarded. A consensus sequence was determined from the sequences which aligned together with 92% similarity to reduce the number of OTUs in the tree and each contig was annotated with its respective number of sequences. All the sequences were related to the *Halobacteriaceae* family, a group of extremely halophilic, aerobic *Archaea*. The majority of them were not closely related to any known species from the GenBank database. Specifically, out of 530 archaeal sequences, only 64 showed >94% similarity with the recorded entries in the GenBank.

**Figure 2:**
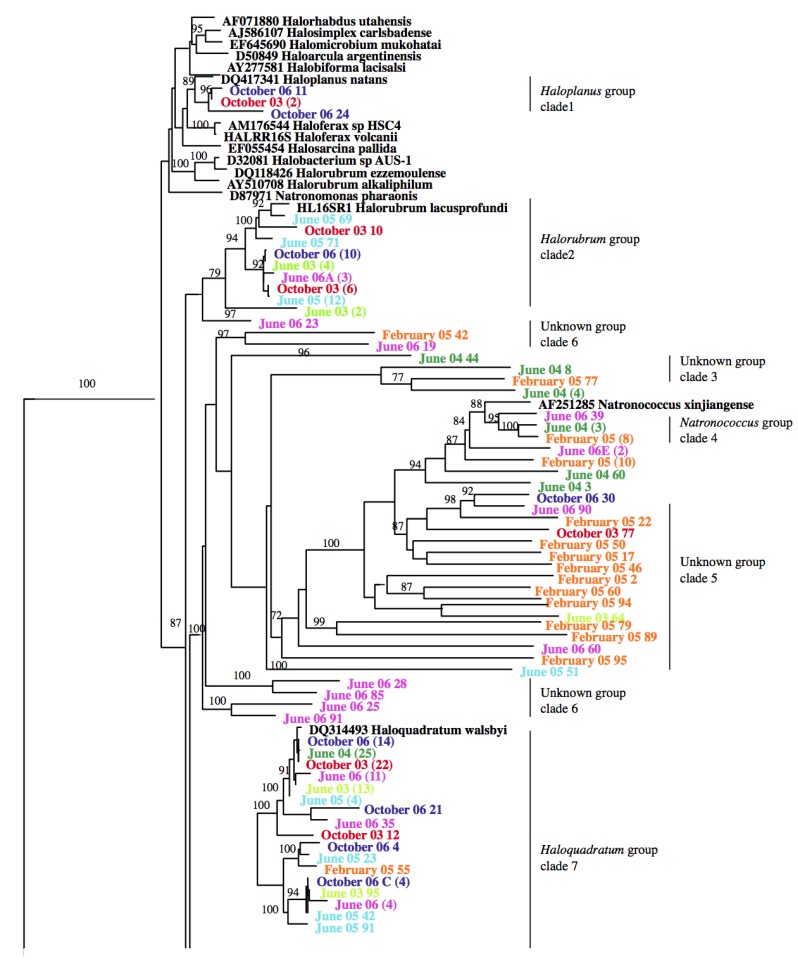

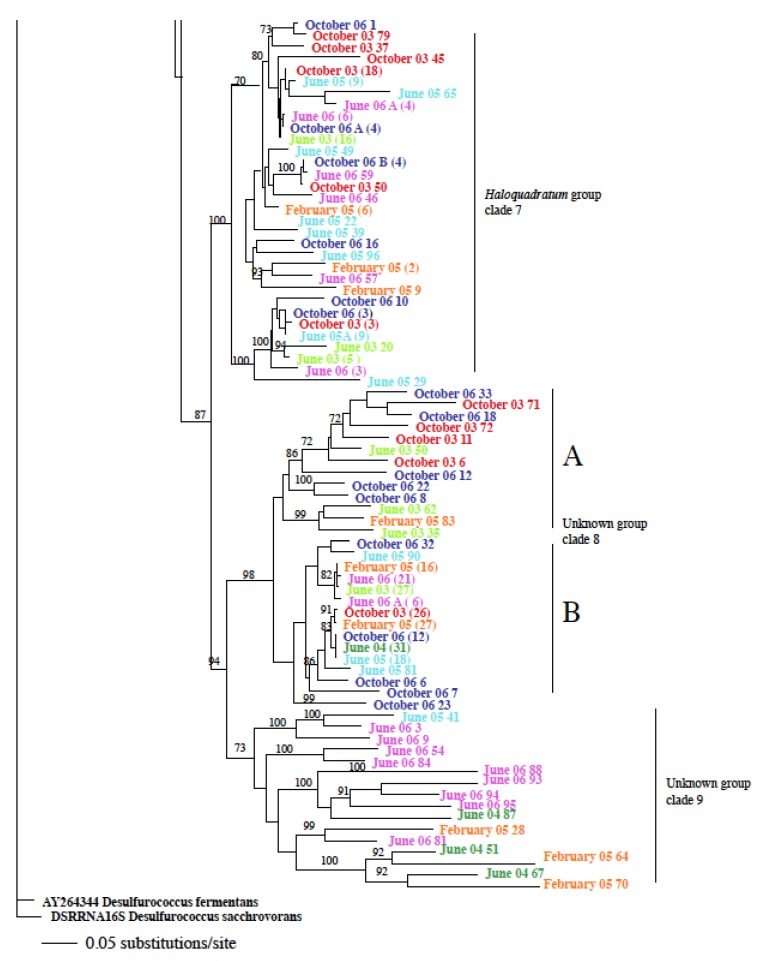
Phylogenetic tree constructed with all of the archaeal sequences retrieved from 16S rRNA gene clone libraries from north arm of Great Salt Lake. Numbers in parentheses indicate the number of sequences in the contig. The known genera from the database were clustered together forming two subclusters. *Halorhabdus*, *Halosimplex*, *Halomicrobium*, *Haloarcula*, *Halobiforma* and *Haloplanus* were all phylogenetically related forming one subcluster while *Haloferax*, *Halosarcina*, *Halobacterium*, *Natronomonas*, and *Halorubrum* formed another sub-cluster. One species of *Halorubrum* (*Hrr. lacusprofundi*) formed the remaining clade.

The archaeal community structure was compared at different time intervals using phylogenetic analysis (Figure 2). It revealed that there were a total of 9 clusters or clades, out of which only four clades showed relatively close identity with known organisms. The latter were three small clades of a *Haloplanus* related group (4 clones) (October 2003 and October 2006), a *Halorubrum* related group (41 clones) (June 2003, June 2005, June 2006, October 2003 and October 2006), a *Natronococcus* related group (14 clones) (June 2004, June 2006 and February 2005), and a large clade of the clones related to *Haloquadratum* (237 clones) (June 2003, June 2004, June 2005, June 2006, February 2005, October 2003 and October 2006) (Figure 2). The remaining five clades did not show close similarity with any of the known groups. The *Haloquadratum* and the unknown group of clade 8B (190 clones) were present in all samples. Thus phylogenetic analysis revealed some temporal variation in the diversity, but no interrelationship was observed between sampling dates and phylogenetic diversity (Figure 2).

**Figure 3:**
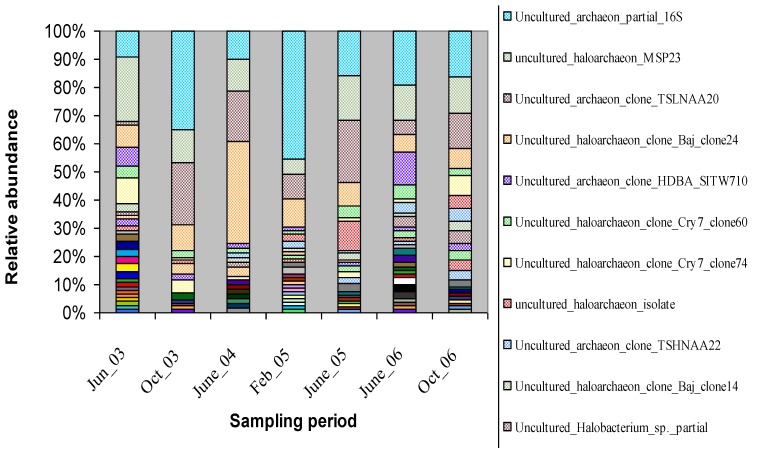
Relative abundance of archaeal community obtained from 16S rRNA gene sequencing in each sampling period. Legends with major relative abundances (>5%) are shown in the figure.

The relative abundances of the archaea were plotted against the sampling period using the RDP10 Bayesian classifier (Figure 3). The bar graph showed that most abundant groups are uncultured archaeon, uncultured haloarchaeon MSP23, uncultured haloarchaeon TSLNAA20, and Baj clone24. These communities were dominant throughout the sampling period except uncultured haloarchaeon TSLNAA20, which was a minor community in June 2003. Also, the abundance of uncultured haloarchaeon Baj clone 24 was almost stable throughout each sampling but increased three fold in June 2004. However, the diversity of minor communities changed in each sampling period.

**Figure 4:**
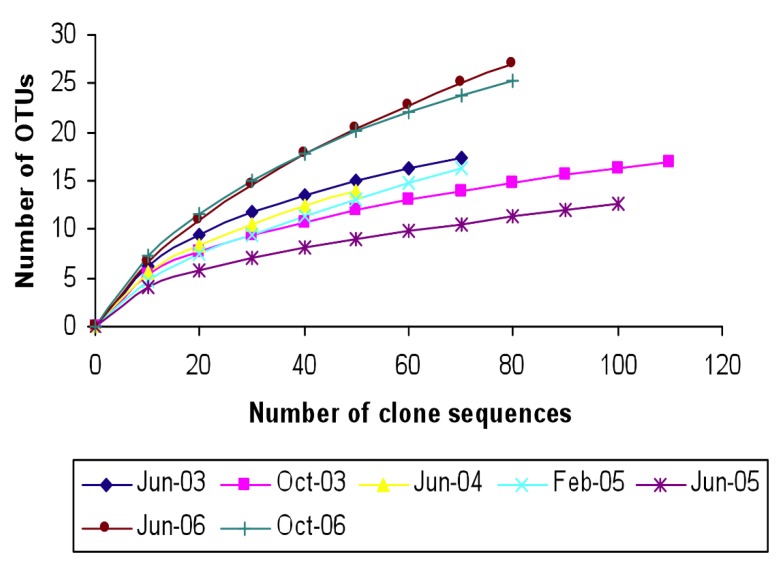
Rarefaction curves (95% similarity cut off) indicating community taxon richness based on 16S rRNA gene sequences of archaea obtained from the north arm of Great Salt Lake.

Figure 4 shows the rarefaction curves generated for the archaeal 16S rRNA genes in the clone libraries from Great Salt Lake samples. Rarefaction analysis was done using 95% similarity cutoff of the clones. The June 2006 and October 2006 had the highest number of OTUs (~25) whereas June 2005 and June 2004 had the lowest number of OTUs (12–14). Rarefaction curves for all the samples with the exception of June 2006 and October 2006 reached the saturation level, which suggests that the sampling efforts were sufficient. The rarefaction curves for June 2006 and October 2006, however, did not level off which suggests that more sampling would have revealed more diversity.

### 3.3. Relatedness of Cloned Libraries and the Influence of Environmental Factors

Web LIBSHUFF v0.96 was used to compare the different clone libraries. Pairwise comparisons of each library to every other library by using ∫-LIBSHUFF revealed that some libraries were distinct from each other whereas some libraries were similar to the other libraries. The archaeal community of Great Salt Lake in June 2003 was significantly different from the June 2004, June 2005, and June 2006 communities (Supplementary Table 1). The October 2003 and October 2006 communities were not significantly different with *p*-values greater than 0.025 while the February 2005 (the only winter sample) community was significantly different from June 2003, June 2006, and October 2006 communities. June 2004, June 2005, and the October 2003 communities were similar to the February 2005 community (Supplementary Table 1).

**Figure 5:**
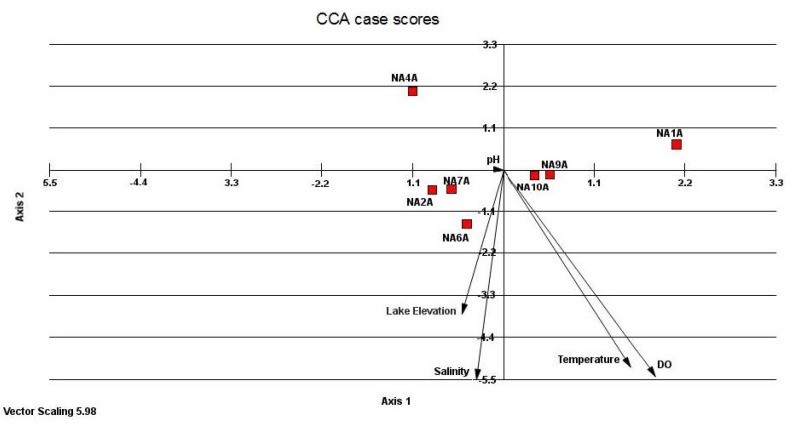
Canonical Correspondence Analysis ordination diagram with relative abundance of seven archaeal communities (squares) and environmental variables (arrows). The cumulative percentage for axis 1 and axis 2 are 26.55 and 33.13 respectively. The seven clone libraries are NA1A (June-2003) NA2A (October-2003), NA4A (June-2004), NA6A (February-2005), NA7A (June-2005) NA9A (June-2006), NA10A (October-2006).

Correlation coefficients between environmental variables (temperature, salinity, pH, DO, and lake elevation) and two CCA axes (Supplementary Table 2) indicated that axis 1 and axis 2 have strongest correlation with the salinity (highest coefficients 1.49 and −0.425). Canonical correspondence analysis (CCA) of five environmental factors and relative abundance of seven clone libraries (Figure 5) indicated that some communities such as NA2A (October 2003), NA6A (February 2005), NA7A (June 2005) were influenced by lake elevation and salinity whereas communities such as NA9A (June 2006) and NA10A (October 2006) were influenced by DO and temperature. However, the locations of these communities were not close to the arrow pointer indicating that the influence was not very strong. The NA4A (June 2004) and NA1A (June 2003) libraries were not at all affected by environmental conditions. Thus, there was no strong correlation found among the communities and environmental factors.

## 4. Discussion

Great Salt Lake is a terminal lake, and thus the lake elevation is a function of precipitation and evaporation, which varies significantly over time (Table 1). When lake levels change as a function of freshwater input, salinity decreases [17], or freshwater streaming changes the compartmentalization of the water column. One would expect to see shifts in microbial communities annually in response to fluctuating precipitation levels, however, we did not observe this connection (Figure 5). In fact, shifts in community structure do not appear to track with any one environmental parameter, including temperature and salinity. Also, some parameters, such as DO and pH, were stable in the shallow north arm, and therefore, these have little impact on changing composition of the microorganisms present. Even salinity, though changing solubility in response to sampling temperature, remains at saturation. A recent study in Australia showed that the relative abundances of *Haloquadratum* were correlated with potassium, magnesium and sulfate, but not sodium or chloride [33]. This begs the question whether other ions in the Great Salt Lake may impact microbial life at the north arm where sulfate, for example, is unusually high as reviewed in [17].

This is in stark contrast to a study from the south arm examining a deep, vertical transect over a salinity and DO gradient where a strong halocline exists [14]. At this sampling site, where heavy concentrated brine forms a layer beneath meters of less saline water, these parameters did influence phylogenetic diversity and the composition of communities. Great Salt Lake is a dynamic ecosystem, but perhaps the isolated north arm is less so, maintaining significant stability of environmental conditions over time.

What other factors impacting microbial diversity should we consider in this extreme environment? We acknowledge the presence of invertebrate grazers, brine flies and brine shrimp, that can impact microorganisms. These may affect the microbial communities of the south arm greatly, but their virtual absence in the north arm suggests little impact in the area. Some shrimp hatch here from cysts blown in, but they do not thrive. Brine fly larvae cannot survive in the salt-saturated brine. 

A more significant player in managing the microbial community profiles could be the halo-viruses [9]. Since *Archaea* species outnumber those in the *Bacteria* domain in this salt-saturated part of Great Salt Lake, we would expect a larger impact on archaeal cells. Indeed, viruses may be responsible for the CFU count variation that we observe (Table 1). If cultivatable strains in one community are susceptible to viral infection, then we should see CFUs go down. If vulnerable strains are non-cultivatable, then CFUs may go up. Not only are viral particles observed in great numbers in this lake, many more could be silent within lysogenic bacteria or archaea as has been observed in a Florida estuary [34]. We have no way to measure their impact, but we must present this caveat: Viruses in an extreme ecosystem like Great Salt Lake, where predators are limited, may be a driving force in shifting the populations of specific genera.

The data presented here highlight that the lake is an unexplored reservoir of many novel phylotypes. Phylogenetic analysis of the Great Salt Lake archaeal community showed that the majority of the sequences were from uncultured taxa similar to that observed in other hypersaline lakes such as Solar Lake, Egypt [35], athalassohaline lakes in the Atacama desert, Chile [36], and Lake Chaka in Northern China [37]. The above studies of the hypersaline habitats have indicated that there are many novel phylotypes within the family *Halobacteriaceae*. In our phylogenetic analyses we used a consensus sequence set at 92% similarity in order to decrease the size of the tree. The phylogenetic tree (Figure 2) showed that known taxa of *Halobactericeae* formed one clade suggesting that there is not much difference in taxa at the 92% similarity level (family level). However, this indicates that even within a single clade of our phylogenetic tree, clones can be very divergent at the species level.

We found a *Salinibacter* related group as the most abundant member of the domain *Bacteria* in Great Salt Lake, which was 97%–99% similar to *Salinibacter ruber*. In crystallizer ponds with 30%–37% salts, the *Salinibacter* group was detected with fluorescent *Salinibacter* specific probe using *in situ* hybridization. This group constituted 5%–25% of the total prokaryotic community of the saltern ponds [38]. A similar population is expected in the north arm with 27% salinity. GenBank analysis also occasionally showed the presence of *Halomonas* and an uncultured halophilic Eubacterium (data not shown).

Analysis of 16S rRNA archaeal sequences via cloning imply that halophilic archaeal sequences belonging to *Haloquadratum* like group [39,40] were the dominant archaeal sequences in most of the communities analyzed. Of the 530 clones analyzed, 237 belonged to this cluster (Clade 7). Presence of this group in all times implies that it is capable of adapting to seasonal fluctuations. The presence of *Haloquadratum* like group, has also been detected in other hypersaline lake [41,42]. Additionally, Great Salt Lake also harbors *Halorubrum*, *Natronococcus* and *Haloplanus*. *Halorubrum* and *Natronococcus* were detected with both the culture–independent and dependent studies [43].

## 5. Conclusions

The idea that microbial communities change over time is a well-accepted paradigm, however, genetic studies tend to take the snapshot approach, analyzing the 16S rRNA gene profiles in a sample from a single day [44]. Looking at these changing community profiles over seasons and over years, impress upon us the changing biochemical capacities of a limited environment such as a terminal lake [35,44,45]. The data presented above show shifts in microbial populations, in terms of abundance and composition over time, but these are not tied to the shifts of a single environmental parameter. This study highlights the complexity of a “simple” ecosystem such as the salt-saturated brine in the north arm of Great Salt Lake.

## Supplementary Files

Supplementary File 1

## References

[1] Cannon J.S., Cannon M.A., Gwynn J.W. (2002). The Southern Pacific Railroad Trestle—Past and present. Great Salt Lake, an Overview of Change.

[2] Great Salt Lake, Utah. http://ut.water.usgs.gov/greatsaltlake/index.html.

[3] Crosman E.T., Horel J.D. (2009). MODIS-derived surface temperature of the Great Salt Lake. Remote Sens. Environ..

[4] Post F.J. (1977). The microbial ecology of the Great Salt Lake. Microb. Ecol..

[5] Prosser J.I., Bohannan B.J.M., Curtis T.P., Ellis R.J., Firestone M.K., Freckleton R.P., Green J.L., Green L.E., Killham K., Lennon J.J. (2007). The role of ecological theory in microbial ecology. Nat. Rev. Microbiol..

[6] Raes J., Bork P. (2008). Molecular eco-systems biology: Towards an understanding of community function. Nat. Rev.Microbiol..

[7] Gonzalez A., King A., Robeson M.S., Song S., Shade A., Metcalf J.L., Knight R. (2012). Characterizing microbial communities through space and time. Curr. Opin. Biotechnol..

[8] Brock T.D. (1966). Principles of Microbial Ecology.

[9] Baxter B., Mangalea M., Willcox S., Sabet S., Nagoulat M.-N., Griffith J., Ventosa A., Oren A., Ma Y. (2011). Haloviruses of Great Salt Lake: A model for understanding viral diversity. Halophiles and Hypersaline Environments.

[10] Konopka A. (2006). Microbial ecology: Searching for principles. Microbe.

[11] Pace N.R. (1997). A molecular view of microbial diversity and the biosphere. Science.

[12] Klepac-Ceraj V., Hayes C.A., Gilhooly W.P., Lyons T.W., Kolter R., Pearson A. (2012). Microbial diversity under extreme euxinia: Mahoney Lake, Canada. Geobiology.

[13] Wilhelm R.C., Radtke K.J., Mykytczuk N.C.S., Greer C.W., Whyte L.G. (2012). Life at the wedge: The activity and diversity of Arctic ice wedge microbial communities. Astrobiology.

[14] Meuser J.E., Baxter B.K., Spear J.R., Peters J.W., Posewitz M.C., Boyd E.S. (2013). Contrasting patterns of community assembly in the stratified water column of Great Salt Lake, Utah. Microb. Ecol..

[15] Parnell J., Rompato G., Crowl T.A., Weimer B.C., Pfrender M.E. (2011). Phylogenetic distance in Great Salt Lake microbial communities. Aquat. Microb. Ecol..

[16] Weimer B.C., Rompato G., Parnell J., Gann R., Ganesan B., Navas C., Gonzalez M., Clavel M., Albee-Scott S. (2009). Microbial biodiversity of Great Salt Lake, Utah. http://digitalcommons.usu.edu/cgi/viewcontent.cgi?article=1311&context=nrei.

[17] Baxter B., Litchfield C., Sowers K., Griffith J., Dassarma P., Dassarma S., Gunde-Cimerman N., Oren A., Plemenitaš A. (2005). Microbial diversity of Great Salt Lake. Adaptation to Life at High Salt Concentrations in Archaea, Bacteria, and Eukarya.

[18] Great Salt Lake—Lake Elevations and Elevation Changes. http://ut.water.usgs.gov/greatsaltlake/elevations/index.html.

[19] Gibbons N.E., Norris J.R., Ribbons D.W. (1969). Chapter VIII: Isolation, growth and requirements of halophilic bacteria. Methods in Microbiology.

[20] Litchfield C.D., Gillevet P.M. (2002). Microbial diversity and complexity in hypersaline environments: A preliminary assessment. J. Ind. Microbiol. Biotechnol..

[21] Litchfield C.D., Irby A., Vreeland R.H. (1998). The microbial ecology of solar salt plants. Microbiology and Biogeochemistry of Hypersaline Environments.

[22] Suzuki M., Rappé M.S., Giovannoni S.J. (1998). Kinetic bias in estimates of coastal picoplankton community structure obtained by measurements of small-subunit rRNA gene PCR amplicon length heterogeneity. Appl. Environ. Microbiol..

[23] Litchfield C.D., Sikaroodi M., Gillevet P.M. (2006). Characterization of natural communities of halophilic microorganisms. Methods Microbiol..

[24] Lane D.J. (1991). 16S/23S rRNA sequencing. Nucleic Acid Techniques in Bacterial Systematics.

[25] Altschul S.F., Madden T.L., Schäffer A.A., Zhang J., Zhang Z., Miller W., Lipman D.J. (1997). Gapped BLAST and PSI-BLAST: A new generation of protein database search programs. Nucleic Acids Res..

[26] Huber T., Faulkner G., Hugenholtz P. (2004). Bellerophon: A program to detect chimeric sequences in multiple sequence alignments. Bioinformatics.

[27] Thompson J.D., Gibson T.J., Plewniak F., Jeanmougin F., Higgins D.G. (1997). The ClustalX windows interface: Flexible strategies for multiple sequence alignment aided by quality analysis tools. Nucleic Acids Res..

[28] Swofford D.L. (2001). PAUP*: Phylogenetic Analysis Using Parsimony, Version 4.0.

[29] LIBSHUFF software v0.96.

[30] Singleton D.R., Furlong M.A., Rathbun S.L., Whitman W.B. (2001). Quantitative comparisons of 16S rRNA gene sequence libraries from environmental samples. Appl. Environ. Microbiol..

[31] Gillevet P., Sikaroodi M., Keshavarzian A., Mutlu E.A. (2010). Quantitative assessment of the human gut microbiome using multitag pyrosequencing. Chem. Biodivers..

[32] Analytical Rarefaction software v1.3.

[33] Podell S., Emerson J.B., Jones C.M., Ugalde J.A., Welch S., Heidelberg K.B., Banfield J.F., Allen E.E. (2014). Seasonal fluctuations in ionic concentrations drive microbial succession in a hypersaline lake community. ISME J..

[34] Cochran P.K., Paul J.H. (1998). Seasonal abundance of lysogenic bacteria in a subtropical estuary. Appl. Environ. Microbiol..

[35] Cytryn E., Minz D., Oremland R.S., Cohen Y. (2000). Distribution and diversity of archaea corresponding to the limnological cycle of a hypersaline stratified lake (Solar Lake, Sinai, Egypt). Appl. Environ. Microbiol..

[36] Demergasso C., Casamayor E.O., Chong G., Galleguillos P., Escudero L., Pedros-Alio C. (2004). Distribution of prokaryotic genetic diversity in athalassohaline lakes of the Atacama Desert, Northern Chile. FEMS Microbiol. Ecol..

[37] Jiang H., Dong H., Zhang G., Yu B., Chapman L.R., Fields M.W. (2006). Microbial diversity in water and sediment of Lake Chaka, an athalassohaline lake in northwestern China. Appl. Environ. Microbiol..

[38] Antón J., Rossello-Mora R., Rodríguez-Valera F., Amann R. (2000). Extremely halophilic bacteria in crystallizer ponds from solar salterns. Appl. Environ. Microbiol..

[39] Burns D.G., Camakaris H.M., Janssen P.H., Dyall-Smith M.L. (2004). Cultivation of Walsbyʼs square haloarchaeon. FEMS Microbiol. Lett..

[40] Burns D.G., Janssen P.H., Itoh T., Kamekura M., Li Z., Jensen G., Rodríguez-Valera F., Bolhuis H., Dyall-Smith M.L. (2007). *Haloquadratum walsbyi gen*. nov., sp. nov., the square haloarchaeon of Walsby, isolated from saltern crystallizers in Australia and Spain. Int. J. Syst. Evol. Microbiol..

[41] Burns D., Camakaris H., Janssen P., Dyall-Smith M. (2004). Combined use of cultivation-dependent and cultivation-independent methods indicates that members of most haloarchaeal groups in an Australian crystallizer pond are cultivable. Appl. Environ. Microbiol..

[42] Ochsenreiter T., Pfeifer F., Schleper C. (2002). Diversity of Archaea in hypersaline environments characterized by molecular-phylogenetic and cultivation studies. Extremophiles.

[43] Almeida-Dalmet S. (2011). A Study of Microbial Diversity in the North Arm of Great Salt Lake. Ph.D. Thesis.

[44] Venter J.C., Remington K., Heidelberg J.F., Halpern A.L., Rusch D., Eisen J.A., Wu D., Paulsen I., Nelson K.E., Nelson W. (2004). Environmental genome shotgun sequencing of the Sargasso Sea. Science.

[45] Demergasso C., Escudero L., Casamayor E.O., Chong G., Balague V., Pedros-Alio C. (2008). Novelty and spatio-temporal heterogeneity in the bacterial diversity of hypersaline Lake Tebenquiche (Salar de Atacama). Extremophiles.

