# Supplementary material for: Temporal Study of the Microbial Diversity of the North Arm of Great Salt Lake, Utah, U.S
Source: Microorganisms. 2015 Jul 2;3(3):310–26. doi: 10.3390/microorganisms3030310 (PMC5023243; doi:10.3390/microorganisms3030310)
Supplement: Supplementary File 1 [file microorganisms-03-00310-s001.docx]

**Supplementary Information**

**Table S1.** ∫-LIBSHUFF analysis. A comparison of 16S rRNA gene sequences from seven archaeal libraries from the north arm of Great Salt Lake based on *p* ^§^ values.

|  | **JUN03** | **OCT03** | **JUN04** | **FEB05** | **JUN05** | **JUN06** | **OCT06** |
| --- | --- | --- | --- | --- | --- | --- | --- |
| JUN03 | - | 0.001 | 0.001 | 0.006 | 0.001 | 0.001 | 0.022 |
| OCT03 | 0.001 | - | 0.003 | 0.441 | 0.554 | 0.001 | 0.14 |
| JUN04 | 0.001 | 0.001 | - | 0.002 | 0.244 | 0.001 | 0.013 |
| FEB05 | 0.001 | 0.279 | 0.474 | - | 0.574 | 0.001 | 0.001 |
| JUN05 | 0.135 | 0.802 | 0.635 | 0.540 | - | 0.001 | 0.012 |
| JUN06 | 0.252 | 0.001 | 0.139 | 0.001 | 0.011 | - | 0.097 |
| OCT06 | 0.027 | 0.449 | 0.006 | 0.001 | 0.305 | 0.001 | - |

^§^ The values indicate the probabilities that the compositions of the libraries were different, calculated by using the LIBSHUFF program. *p* *=* 0.001.

**Table S2.** Correlation coefficients between environmental variables and the first two CCA axes.

| **Variables** | **Axis 1** | **Axis 2** |
| --- | --- | --- |
| pH | 0 | 0 |
| DO ^§^ | −0.849 | −0.12 |
| Temperature | −0.772 | 0.364 |
| Lake elevation | −0.007 | 0.294 |
| Salinity | 1.499 | −0.425 |

^§^ DO—Dissolved Oxygen.
